# Factors associated with burnout among healthcare providers in a rural context, South Africa

**DOI:** 10.4102/phcfm.v16i1.4163

**Published:** 2024-01-23

**Authors:** Alexandra C. Moses, Abigail R. Dreyer, Lesley Robertson

**Affiliations:** 1 School of Public Health, Faculty of health Sciences, University of the Witwatersrand, Johannesburg, South Africa; 2 Department of Family Medicine and Primary Care, Faculty of Health Sciences, University of the Witwatersrand, Johannesburg, South Africa; 3 Department of Psychiatry, Faculty of Health Sciences, University of the Witwatersrand, Johannesburg, South Africa

**Keywords:** burnout, healthcare workers, South Africa, rural, primary health care, sub-Saharan Africa, mental health, occupational stress, public health

## Abstract

**Background:**

Healthcare providers (HCPs) are at risk of burnout in sub-Saharan Africa. However, there is little research in rural and primary care settings.

**Aim:**

To evaluate burnout and its associated factors among public sector HCPs in South Africa.

**Setting:**

Primary health care clinics, community health centres and district hospitals in Nkomazi Local Municipality, Mpumalanga province.

**Methods:**

Quantitative study design using a cross-sectional survey. Healthcare providers (*n* = 1139) working in Nkomazi Local Municipality were invited to participate. Burnout was assessed using the Maslach Burnout Inventory. A demographic and occupational questionnaire, the General Help-Seeking Questionnaire and the Health and Safety Executive Indicator Tool were used.

**Results:**

A total of 302 HCPs, between 23 and 61 years, mostly female (*n* = 252; 83.44%) and nurses (*n* = 235; 77.81%) participated. High burnout was observed for Emotional Exhaustion (median score 26 [IQR {interquartile range}: 34–16]) and Personal Accomplishment (median score 29 [IQR: 34–25]). Most participants (*n* = 215; 71.19%) would seek help if they had an emotional problem. Bivariate analysis revealed significant associations between workplace demands, control, management support, peer support, relationships, role and change with all subscales of burnout. Multivariate regression analysis found that Personal Accomplishment improved by 0.49 (95% CI: 0.10–0.89) for every point in improved work demands, by 0.84 (95% CI: 0.01–1.67) for every point towards improved management support and by 1.19 (95% CI: 0.48–1.90) for every point towards having an improved role.

**Conclusions:**

During 2022, HCPs working in a rural area in South Africa displayed high levels of burnout for Emotional Exhaustion and Personal Accomplishment but not for Depersonalisation.

**Contributions:**

Improvements in work demands, managerial support and role clarity may reduce burnout among HCP in a rural, primary care setting.

## Introduction

The World Health Organization defines burnout as being ‘a syndrome conceptualized as resulting from chronic workplace stress that has not been successfully managed’.^1^ It is characterised by three dimensions: feelings of energy depletion or exhaustion, increased mental distance from one’s job or feelings of negativism or cynicism related to one’s job, and reduced professional efficacy.^1,2^ In May 2019, the World Health Organization included burnout as an occupational phenomenon in the International Classification of Diseases.^1^

Workplace stress is defined as the response to work demands and pressures, which exceed knowledge, capacity and abilities, challenging the ability to cope.^3^ In sub-Saharan Africa, healthcare providers (HCPs) are susceptible to high levels of burnout as a result of occupational stress from staff shortages, insufficient resources and supplies, high workload and hours worked.^4^ Burnout has implications for the well-being of HCPs, affecting productivity, performance and staff turnover.^4^ Low levels of well-being result in absenteeism, exacerbating staff shortages.^5^ The needs of HCPs have to be supported to prevent resignations as a result of burnout.^6^ This is important on a larger scale in the recruitment and retention of the healthcare work force.^6^

There is little research on the factors associated with burnout in the workplace within low- and middle-income countries.^5^ The current context justifies the need for this study as HCPs in South Africa are faced with the management of a quadruple burden of disease. More attention needs to be given to HCP burnout in a rural, district-level setting so that the factors associated with burnout are understood, thereby informing workplace strategies to support HCPs.^4,5^

### Aim and objectives

The aim of the research was to describe the experience of burnout and to examine associated individual and workplace factors among HCPs employed by the Department of Health (DOH) within a rural context (location masked for blind review) in 2022. The objectives of the study were to describe individual and workplace factors, to describe the experience of burnout according to scores on Emotional Exhaustion, Depersonalisation and Personal Accomplishment, and to examine the association of burnout with individual and workplace factors among HCPs employed by the DOH within a rural context (location masked for blind review) in 2022.

## Research methods and design

### Study design

A quantitative study design using a cross-sectional survey was employed. A descriptive, analytical approach was used to identify the factors associated with burnout among rural HCPs.

### Setting

The research was conducted in the Nkomazi Local Municipality, which is situated in the eastern part of the Mpumalanga province. This local municipality has 26 primary health care (PHC) clinics, 6 community health centres (CHCs) and 2 district hospitals. The district hospitals render level-one hospital services and include a 350-bed facility and a 230-bed facility.^7^ These healthcare facilities serve a population of 410 907 people comprising 103 965 households.^7^

### Study population and sampling strategy

The study population comprised HCPs employed by the DOH and working in the district hospitals, the CHCs and PHC clinics in the local municipality in 2022. The total number of HCPs employed within this area was 1139 according to human resource data from March 2021. The sample size needed for 95% confidence level was 287, assuming that the population was 1139, expected frequency was 50% and confidence limits were 5%. The number of participants was 302, translating to a response rate of 26.5% according to the HCP population size. Two hospitals, 3 CHCs and 13 PHC clinics were visited to recruit participants. The sites were utilised because of ease of access via public transport for the field workers and until the response rate needed for 95% confidence level was reached.

#### Inclusion criteria

Participants were HCPs 18 years and older, employed by the DOH (whether temporary or permanently employed), working in the district hospitals and the selected CHCs and PHC clinics in the local municipality in 2022, regardless of the duration of employment. Healthcare providers included participants as according to the definition in the *National Health Act 61 of 2003*.^8^ Participants included community service professionals.

#### Exclusion criteria

Participants who are HCPs, who are currently registered as students and/or who were younger than 18 years were excluded.

### Participant recruitment

Participant recruitment took place from April to September 2022. The Heads of Departments (HODs) and clinic facility managers were contacted in the recruiting of participants via email and telephone in April 2022. They were requested to assist in distributing the questionnaire to their teams, inviting all clinical staff to respond voluntarily. Follow-up with the HODs and clinic facility managers was conducted on a monthly basis for the period of 4 months. Recruitment of participants was also conducted via social media on Facebook groups and via WhatsApp messages to personal contacts of the principal investigator. Because of the poor response rates from April to August, two field workers (third-year health sciences students who were local to the area) assisted with in-person, voluntary recruitment from 15 August to 08 September 2022. They were responsible for recruiting participants to complete the self-administered questionnaires and worked under supervision of the principal investigator. They visited 18 sites (2 hospitals, 3 CHCs and 13 PHC clinics) within the municipality.

### Data collection

Data were collected via a self-administered questionnaire. Participants completed the questionnaire online on their smart phone or tablet; the field worker distributed the link for the online questionnaire and hardcopies of the questionnaire, printed from the electronic version.

In addition to demographic items (age, gender, designation, years of employment, etc.), the questionnaire contained three standardised assessments, namely the Maslach Burnout Inventory – Human Services Survey (MBI-HSS), the General Help-Seeking Questionnaire (GHSQ) and the Health and Safety Executive (HSE) Indicator Tool.

Burnout was assessed using the MBI-HSS, which assessed three core aspects of the burnout syndrome: Emotional Exhaustion (feelings of being emotionally overextended and exhausted by one’s work), Depersonalisation (an unfeeling and impersonal response towards the recipients of one’s service, care or treatment) and Personal Accomplishment (feelings of competence and success in one’s work).^2^

The GHSQ, a validated scale consisting of future intentions to seek help from a list of culturally relevant sources (both formal and informal), was used to measure help-seeking intentions.^9^ The GHSQ uses a matrix format that can be modified according to purpose and need.^9^

The HSE has adopted a standard-based approach to addressing work-related stress and has defined six management standards that represent aspects of work.^10^ The HSE Indicator Tool is based on desired standards being identified and comparing them against actual or current standards.^10^ If poorly implemented, the six management standards identified are associated with lower levels of health, productivity and well-being and with increased sickness absence.^10^ The six management standards are demands, control, support, relationships, role and change.^10^

### Data analysis

Data were entered through the REDCap platform, hosted by the University of Witwatersrand.^11,12^ Data were exported directly from REDCap to STATA V.16.1 for analysis. Categorical variables collected were coded numerically and continuous variables were cleaned and standardised in terms of units measured in STATA V.16.1 in preparation for analysis.

Descriptive statistics were used to summarise the characteristics of the data set. A bivariate analysis (an analysis conducted to examine the relationship between two variables) was used to examine the relationship between each individual and workplace variable with each of the three burnout subscales of Emotional Exhaustion, Depersonalisation and Personal Accomplishment. The burnout subscales were the dependent variable. Statistically significant associations were examined using the crude estimate of 0.25. Multiple linear regression was used to examine any factors associated with burnout outcomes. A multiple linear regression model was built to account for confounding, using stepwise variable selection. A significance of 5% was used for statistical significance testing.

### Ethical considerations

Ethical clearance was obtained from the Institutional Review Board at University of the Witwatersrand’s Human Research Ethics Committee (HREC) on 24 March 2022 with ethical application reference number M2111131, and permission was received to conduct research at the study site from the Mpumalanga Provincial Health Research Committee (PHRC) on 07 April 2022 with reference number MP_202201_003. Letters of support were received from the CEOs of the hospitals (on 08 November 2021 and 13 August 2021) and the District Manager (on 23 July 2021) to conduct research in the area. The field worker was given written, signed permission by the principal investigator to recruit participants on her behalf. All questionnaires were anonymous and labelled according to participant numbers. Consent was obtained from all participants prior to their enrolment in the study by virtue of the voluntary nature of completing the questionnaire.

## Results

### Individual factors

The number of participants was 302 (Table 1) translating to a response rate of 26.5% according to the HCP population size. Most participants were nurses (*n* = 235; 77.81%), with ages ranging from 23 to 61 years with a median age of 38.5 years (IQR [interquartile range]: 45–32), with the majority of participants being South African citizens (*n* = 295; 97.7%), black African (*n* = 294; 97.4%), female (*n* = 252; 83.44%) and having tertiary-level education (*n* = 296; 98.01%). Most of the participants lived locally (*n* = 215; 71.19%); those who had moved to the area had done so on average 2 years (IQR: 5 – 2 months) prior to the study. Half of the participants were in a long-term, stable relationship (*n* = 157; 51.99%) and two-thirds of participants had children (*n* = 191; 63.25%). Over three-quarters of the participants (*n* = 236; 78.15%) reported to have a religious affiliation.

**TABLE 1:** Descriptive analysis of individual factors (*n* = 302).

Individual factors	Frequency	%
**Gender**		
Male	50	16.6
Female	252	83.4
**South African citizen**		
Yes	295	97.7
**History of relocation to current workplace**		
Yes	87	28.8
**Level of education**		
High school	3	0.9
Tertiary level	296	98.0
Missing data	3	0.9
**Marital status**		
Single	112	37.1
Married	63	20.9
Living together	94	31.1
Separated	9	2.9
Divorced	7	2.3
Widowed	17	5.6
**Number of children**		
0	111	36.7
1	43	22.5
2	68	35.6
3	43	22.5
4	20	10.5
5+	12	6.3
Missing data	5	1.7
**Religious affiliation**		
Yes	236	78.1
Missing data	6	1.9

The majority of participants (*n* = 215; 71.19%) indicated that it was extremely unlikely that they would not seek help from anyone. Mental health professionals were the most sought-after source of assistance, while traditional healers were the least sought-after source of assistance (Table 2). Summed scores of the GHSQ indicated a median score of 43 (IQR: 49–38) from a maximum score of 77, with higher scores indicating greater help-seeking intentions.

**TABLE 2:** Descriptive analysis results for the General Help-Seeking Questionnaire per domain (*n* = 302).

GHSQ Domain	Descriptive analysis results	
	Median	IQR
Intimate partner	5	6–3
Friend	5	5–3
Parent	5	7–3
Other relative or family member	4	5–3
Mental health professional	5	7–4
Phone helpline	3	5–2
Doctor or GP	4	5–3
Minister or religious leader	4	5–2
Traditional healer or community leader	1	2–1
Not interested to seek help	1	2–1
Would seek help elsewhere	1	4–1

Note: lower bound = 1; upper bound = 7.

GHSQ, General Help-Seeking Questionnaire; IQR, interquartile range.

### Workplace factors

The workplace factors are outlined in Table 3. The participants were fairly evenly spread between those who worked at District Hospital level (*n* = 89; 29.47%), PHC level (*n* = 120; 39.74%) and CHC level (*n* = 91; 30.13%). The majority of participants held permanent positions (*n* = 250; 82.78%), did not engage in shift work (*n* = 261; 82.33%) and had a median number of years of experience of 8 years (IQR: 13–6), with 6 years (IQR: 10–2) of experience in their current work facility. Most (*n* = 261, 82.3%) worked office hours (7 AM – 4 PM), 37 participants (12.3%) did day shifts and only 3 participants (1.0%) did night shifts. Participants worked a minimum of 7 h and maximum of 12 h per day, with the median number of hours worked per day being 8.5 h (IQR: 9–8).

**TABLE 3:** Descriptive analysis of workplace factors (*n* = 302).

Workplace factors	Frequency	%
**Professional category**		
Audiology	2	0.7
Dietetics	6	2.0
Medical practitioners and specialists	5	1.7
Nursing	235	77.8
Occupational therapy	4	1.3
Pharmacy	6	2.0
Physiotherapy	5	1.7
Psychology	2	0.7
Radiography	1	0.3
Social work	26	8.6
Speech and language therapy	2	0.7
Other	6	2.0
Missing data	2	0.7
**Job status**		
Permanent	250	82.8
Contract	49	16.2
Missing data	3	1.0
**Type of facility**		
District hospital	89	29.5
Primary health care clinic	120	39.7
Community healthcare centre	91	30.1
Missing data	2	0.7

The HSE Indicator Tool was used to identify variables associated with work-related stress. As outlined in Table 4, higher scores reflect better working conditions.

**TABLE 4:** Descriptive statistics and percentiles for the Health and Safety Executive Management Standards Indicator Tool (*N* = 302).

Descriptive statistics and percentiles	Demands*	Control	Managerial support	Peer support	Relationships*	Role	Change
Mean	2.99	3.32	3.81	3.81	3.81	4.27	3.52
s.d.	0.70	0.64	0.68	0.59	0.73	0.54	0.77
**Percentiles**							
25%	2.50	3.00	3.40	3.50	3.25	4.00	3.00
50%	2.88	3.33	3.70	3.75	4.00	4.20	3.33
75%	3.63	3.67	4.20	4.00	4.25	4.80	4.00

s.d., standard deviation.

* Scores reversed so that a higher value in the table indicates *less risk* of stress at work, as is the case in the other factors.

**FIGURE 1:**
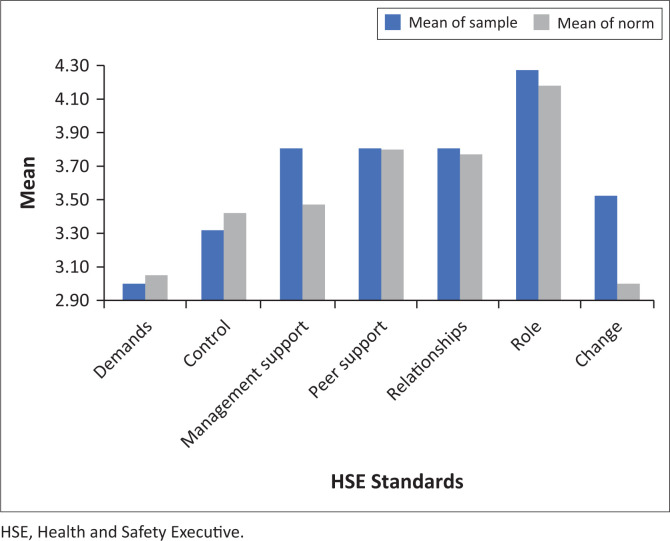
Means of the scores for each standard of the seven-factor Health and Safety Executive Management Standards Indicator Tool and the normative values.

Means of the scores for each standard are illustrated with the normative data in Figure 1.^13^ Scores that were lower than the norm indicate increased work-related stress in the sample as is apparent in the Demands and Control standards.

### Experience of burnout (measured by Maslach Burnout Inventory – Human Services Survey)

**FIGURE 2:**
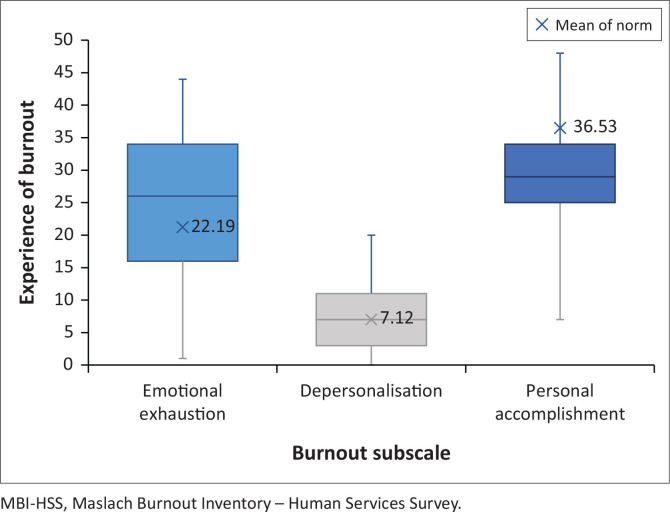
Results of the Maslach Burnout Inventory (*n* = 302).

Figure 2 describes the median and interquartile range, minimum and maximum scores of the burnout subscales. The median was used, as the results for each burnout subscale had a skewed distribution. The median for Emotional Exhaustion was 26 (IQR: 34–16), Depersonalisation was 7 (IQR: 11–2) and Personal Accomplishment was 29 (IQR: 34–25).

Normative data were normally distributed; thus, the mean was used. Normative data for medical professionals indicate that scores for Emotional Exhaustion in this population are higher than the norm of 22.19, Depersonalisation scores were similar to the norm of 7.12, and Personal Accomplishment was lower than the norm of 36.53. The median of each subscale indicates a greater experience of Emotional Exhaustion and decreased Personal Accomplishment in this sample as compared to mean of the normative data.

### Association of burnout with individual and workplace factors

The association between burnout with individual and workplace factors among HCPs employed by the DOH within a rural context in 2022 was examined. Multivariate regression results for individual and workplace factors associated with Emotional Exhaustion, Depersonalisation and Personal Accomplishment are presented in Table 5.

**TABLE 5:** Multivariate regression results for individual and workplace factors associated with Maslach Burnout Inventory scales (*N* = 302).

Individual and workplace factors	Multivariate analysis											
	Emotional exhaustion				Depersonalisation				Personal accomplishment			
	Coef.	95%	CI	*P*	Coef.	95%	CI	*P*	Coef.	95%	CI	*P*
**Individual factors:**												
**Age**	-	-	-	-	-	-	-	-	−0.66	−0.99	−0.32	**< 0.01**
**Gender**												
Male	-	-	-	-	−3.01	−8.87	2.84	0.28	-	-	-	-
**South African citizen**												
Yes	-	-	-	-	-	-	-	-	−0.76	−3.46	1.93	0.56
**History of relocation to current workplace**												
Yes	8.84	−121.24	138.92	0.55	0.24	−6.59	7.07	0.94	-	-	-	-
**Marital status**												
Married	−3.74	−114.12	106.63	0.74	3.27	−3.24	9.79	0.29	0.66	−3.22	4.56	0.72
Living together	14.36	−114.29	143.03	0.39	0.30	−4.00	4.61	0.88	0.12	−2.67	2.92	0.93
Separated	3.00	−132.58	138.60	0.83	2.06	−3.32	7.44	0.41	−2.17	−6.78	2.44	0.34
Divorced	48.19	−221.86	318.25	0.26	4.98	−5.1	15.07	0.29	14.3	5.98	22.62	**< 0.01**
Widowed	28.45	−762.93	819.84	0.73	0.94	−8.48	10.38	0.83	1.05	−4.39	6.50	0.69
**Children**												
Yes	-	-	-	-	0.28	−4.48	5.04	0.90	−1.7	−4.81	1.41	0.27
Number of children	2.75	−70.67	76.17	0.72	-	-	-	-	-	-	-	-
**Religious affiliation**												
Yes	3.33	−123.22	116.55	0.78	0.61	−3.43	4.66	0.74	−1.94	−4.50	0.61	0.13
**Help-seeking intentions**												
GHSQ = 31–50	-	-	-	-	4.91	−4.9	14.73	0.29	-	-	-	-
**Workplace factors:**												
**Type of facility**												
CHC	1.44	−137.85	140.75	0.92	3.96	−3.59	11.53	0.27	5.47	0.38	10.56	**0.04**
**Professional category**												
Pharmacy	-	-	-	-	0.41	−15.3	16.14	0.95	-	-	-	-
**Night shift**												
Yes	-	-	-	-	−5.94	−20.75	8.86	0.39	13.69	3.95	23.44	**0.01**
**Job status**												
Permanent	-	-	-	-	0.34	−4.63	5.31	0.88	-	-	-	-
Contract	-	-	-	-	-	-	-	-	−3.2	−6.02	−0.37	**0.03**
**Total years of experience**	−1.4	−25.20	22.38	0.59	−0.56	−2.27	1.13	0.47	0.36	−0.10	0.83	0.12
**Years of experience in current facility**	-	-	-	-	0.47	−1.18	2.12	0.54	-	-	-	-
**Number hours worked**												
8	−29.68	−282.51	223.15	0.38	−7.81	−17.62	1.99	0.11	-	-	-	-
8.5	-	-	-	-	−9.13	−26.13	7.86	0.26	-	-	-	-
9	−44.09	−296	207.81	0.27	−9.04	−19.79	1.71	0.09	-	-	-	-
12	-	-	-	-	−1.68	−18.24	14.86	0.82	-	-	-	-
**HSE Demands**	0.38	−55.13	55.91	0.94	−0.55	−1.09	−0.01	**0.05**	0.49	0.09	0.89	**0.02**
**HSE Control**	−0.47	−20.06	19.12	0.81	0.17	−0.30	0.65	0.44	0.06	−0.33	0.45	0.74
**HSE Management support**	−4.01	−74.37	66.34	0.60	0.41	−0.75	1.58	0.44	0.83	0.01	1.66	**0.05**
**HSE Peer support**	−1.78	−56.18	52.60	0.75	−0.71	−2.08	0.65	0.27	-	-	-	-
**HSE Relationships**	−4.62	−114.79	105.55	0.69	−0.96	−1.98	0.06	0.06	0.56	−0.05	1.18	0.07
**HSE Role**	8.14	−75.33	91.62	0.43	0.67	−0.58	1.93	0.26	1.19	0.48	1.9	**< 0.01**
**HSE Change**	0.30	−48.3	48.92	0.95	−0.47	−2.08	1.12	0.52	−0.73	−2.01	0.54	0.24

Note: Values in bold were statistically significant (*p* < 0.05).

GHSQ, General Help-Seeking Questionnaire; CHC, community health centres; HSE, Health and Safety Executive; Coef., coefficient.

## Discussion

This research evaluated the severity of burnout and associated individual and workplace factors among HCPs employed by the DOH within a rural context during 2022. The study sample comprised 302 participants, aged between 23 and 61 years, mostly female nurses. High levels of burnout were observed for Emotional Exhaustion and Personal Accomplishment but not for Depersonalisation. Most participants would seek help if they had an emotional problem, most likely from mental health professionals and least likely from traditional healers. On multivariate regression analysis, no individual demographic or workplace factors were significantly associated with Emotional Exhaustion or Depersonalisation. However, Personal Accomplishment increased with an improvement in work demands, managerial support and role understanding.

### Participants

The description of the individual factors indicated that HCPs employed by the DOH within a rural context in 2022 were a stable population, being predominantly culturally and ethnically homogeneous. Although most participants were nursing professionals, allied health professionals and medical practitioners were adequately represented when comparing the study sample to the HCP human resource data from the DOH. This correlates to the study setting as the research was conducted within the rural, PHC setting, where services are predominantly nurse-driven.^14^

The majority of the population were local to the area or moved an average of 2 years prior, with a significant duration of employment, indicating experience. Years of experience working within the sector was found to be associated with higher well-being and may account for the findings of less Emotional Exhaustion than anticipated.^4^

### Experience of burnout

The MBI-HSS scores revealed higher than normative experiences of Emotional Exhaustion and Personal Accomplishment but not for Depersonalisation among study participants. It is difficult to quantitatively compare the data of the MBI-HSS scores to prior studies because the variation in how burnout is defined, assessed and reported.^4^ A systematic review conducted by Owuor et al. examined burnout in nursing professionals in 12 studies across seven African countries (*n* = 2543), with 6 of the studies conducted in South Africa.^15^

It was found that high levels of burnout were recorded among nurses across all dimensions, with the prevalence of Emotional Exhaustion being 66%, Depersonalisation 60% and low Personal Accomplishment 49%.^15^ However, a systematic review of 65 articles in sub-Saharan Africa, 27 of those based in South Africa, by Dubale et al. found the prevalence of Emotional Exhaustion ranging between 12.5% and 65.2%, Depersonalisation between 5% and 57.8% and reduced Personal Accomplishment between 25% and 85.1%.^4^

Dubale et al. found that in sub-Saharan Africa, the highest levels of burnout were recorded among nurses and were associated with unfavourable working conditions, increased job demands and low job satisfaction.^4^ Healthcare providers with less support or resources to manage the increased job demands were found to be associated with high levels of burnout.^4^ These factors and others that emerged from the data that were associated with a higher than normative experience of Emotional Exhaustion and Personal Accomplishment but not for Depersonalisation are explored in subsequent paragraphs.

### Factors associated with an increased experience of burnout

#### Emotional exhaustion

On univariate analysis, many factors were associated with increased Emotional Exhaustion among HCPs in this context, particularly with regard to working conditions including demands, control, managerial support, peer support, relationships, role and change. However, adjustment for confounding factors on multivariate analysis resulted in many of the working conditions and burnout factors negating each other, with no single variable being significantly associated with Emotional Exhaustion. This does not diminish the importance of challenging working conditions being associated with an increased experience of Emotional Exhaustion, as job characteristics inherently influence emotional stress and thus Emotional Exhaustion.^2^

The higher than normative experience of Emotional Exhaustion among the participants of this study is consistent with the previous finding that HCPs working rurally care for a disproportionally greater number of patients because of outmigration of HCPs to urban areas, the private sector and from primary to higher levels of care.^4,6,14,16^ The resulting staff shortages contribute to higher time pressures because of the excessive workloads, which was found by Owuor et al. to increase the experience of Emotional Exhaustion among nurses in Africa.^15^

#### Depersonalisation

Depersonalisation is described by Maslach as ‘negative, cynical attitudes and feelings about one’s patients’, leading to a perception of dehumanisation of others.^2^ Maslach found that the development of Depersonalisation appeared to be related to the experience of Emotional Exhaustion.^2^ However, the MBI-HSS scores revealed that Depersonalisation scores were similar to normative data, thus indicating no increase in Depersonalisation found among study participants.

Dubale et al. found that recurrent night duty and interpersonal conflict among colleagues were predictors of increased Depersonalisation.^4^ It could be that our study did not reveal an increase in Depersonalisation because only three (<1%) participants performed night shift. In addition, as indicated by the scores on the HSE ‘Relationships’ subscale being similar to HSE norms, it appears that interpersonal conflict and unacceptable behaviour in the workplace were being managed appropriately.

Awareness of Depersonalisation by nursing professionals was found in a qualitative study conducted by Hobbs et al., who interviewed 10 nursing professionals on their lived experiences of practising ‘caring presence’ in a rural, public hospital in South Africa.^17^ The shortage of resources, including staff shortages, the lack of time and excessive workloads have been found to result in poor nurse-to-patient ratios in rural areas in South Africa.^17^ Poor nurse-to-patient ratios increase barriers in allowing nurses to practise a caring presence in the treatment of patients.^17^ Nurses in the study by Hobbs et al. were found to be aware of the increased risk of Depersonalisation and the resulting dehumanisation of patients when confronted with an emphasis on productivity and high caseloads in the South African healthcare system, despite strongly expressing against unethical and uncaring nursing actions.^17^

#### Personal accomplishment

Personal Accomplishment is described by Maslach as ‘reflecting a dimension of self-evaluation’, which includes evaluating the job characteristics that influence the resources available to handle the job successfully.^2^ As is well-documented, rural health systems are under-resourced, including in human resources, leading to increased demands and decreased control at work.^6,14,16^ This was reflected in the data as seen by the increased work-related stress when comparing to normative data in the HSE Demands and Control standards in the sample. Low levels of Personal Accomplishment may result from frustrations that come from working in an under-resourced rural health system and the relative inability to change these conditions.^18^ A lack of accomplishment and the occupational stress that this brings may be as a result of a mismatch between the efforts HCPs make and the rewards gained in their occupational environments and efforts represented by the demands placed on HCPs and rewards represented by the returns such as financial compensation, career development opportunities, recognition and esteem.^19^

### Factors associated with a reduced experience of burnout

#### Emotional exhaustion

In the univariate analysis, it was found that being a South African citizen, following a religion and having a married marital status were significantly associated with reduced Emotional Exhaustion. There is a possibility that the social support found in participation in religion or from stable relationships may reduce the experience of Emotional Exhaustion. According to Dubale et al., adequate social support was found to be a protective factor against burnout among HCP.^4^ Increased social support in the forms of being married and being local to the area, thereby having an entrenched network of support, emerged as reducing the experience of Emotional Exhaustion.^4^

In a study by Young and Pakenham with 369 participants in 77 countries, which examined risk and protective factors pertaining to the mental health of aid workers, religiosity was found to be a protective factor.^20^ Bentzen found that humans are more likely to turn to religion for comfort and explanation, particularly when faced with adversity.^21^ Religion is used to reduce emotional distress arising from, in particular, negative and unpredictable situations.^21^ It could be argued that the situation HCPs face in a rural, South African healthcare system could be described as ‘negative and unpredictable’, therefore explaining why religiosity reduced the experience of Emotional Exhaustion.^18,22^

This sample was found to seek help from a variety of sources, with the preferred source of help being mental health professionals. This indicates strong help-seeking skills, with HCPs further utilising their support system, which has been found to be a protective factor in promoting wellness and resilience.^12^ A systematic review by Zaman et al. examined 16 studies from the United Kingdom, the United States, Australia, South Africa, Singapore and South Korea on the barriers and facilitators to seeking help for mental health in doctors.^23^ Facilitators found to increase help-seeking behaviour included positive views about mental health, greater awareness and accessibility to mental health services and supportive supervisors.^23^

Ganasen et al. found that there was a need for improved dissemination of information on mental health among PHC workers as they are the first medical contact in many rural populations and improved mental health literacy could facilitate early recognition of mental illness and appropriate treatment seeking.^24^ The health literacy in this population may be high because of being an HCP and therefore being exposed to appropriate sources in seeking help for mental health support in their employment, which they can then translate into their personal lives.^24^

As a result of this sample being homogeneous with a significant duration of employment, and management support perceived as being higher than normative data, this could account for less Emotional Exhaustion than anticipated, despite the challenging work conditions. Social support includes the perceived support from management. Management support may be perceived as being greater than the norms as some research has argued that rural areas are shaped by more ethnically and culturally homogeneous populations that tend to be more cohesive, which could include greater cohesion between employees and their managers.^25^

In the literature, it has been postulated that HCPs have heterogeneous needs based on specific challenges, predispositions, gender, race, socioeconomic status and other factors, of which managers and leaders will need to be aware to address the challenges and needs in the workplace.^26^ However, as the sample was homogeneous with a significant duration of employment, it could mean that managers are more aware of the challenges and needs of these HCPs.

#### Depersonalisation

The MBI-HSS scores revealed Depersonalisation scores were similar to normative data, thus indicating a reduced experience of burnout on this subscale. Despite postulating that high work demands and time pressure could increase the experience of Depersonalisation, the embeddedness of HCP within the communities in which they work, combined with a strong support system and high help-seeking intentions, may account for the decreased experience of Depersonalisation in this research.^19^ As the majority of participants were local to the study site and had connections existing within their local community, it may be that HCPs share a close relationship with their patients and treat them as if they were family members.^17^ These experiences could be regarded as meaningful and enriching moments, thus decreasing the experience of Depersonalisation.^17^

#### Personal accomplishment

Increased Personal Accomplishment emerged as a variable, which was significantly associated with a number of workplace factors, particularly with regard to an improvement in demands, managerial support and role clarity. Healthcare providers employed by the DOH within this context had a clear understanding of their role within the organisation. This aligns with the implementation of PHC re-engineering goals where an understanding of HCP’s roles in relation to self and others is crucial for a functioning rural healthcare system, especially within the ever-changing ‘task shifting’ that occurs within the PHC system.^6,19^

The necessity of improved managerial support within the rural health system clearly emerged as a mitigating factor to HCPs feeling unhappy about themselves and dissatisfied with their accomplishments at work through management support increasing HCP recognition and esteem.^2,19^ This has been confirmed as one of the reasons for many HCPs wanting to leave the public sector, as was found with medical doctors practising in rural KwaZulu-Natal.^18^

Personal Accomplishment, in HCPs feeling satisfied, passionate, fulfilled and engaged in the communities within their work, can mitigate the experience of working in demanding work conditions caused by high workloads, staff shortages and poor resources.^19^ However, despite HCPs experiencing gratification from their work, improving the experience of Personal Accomplishment does not replace the need for an improvement in working conditions, and this possibly explains why Personal Accomplishment scores on the MBI-HSS were found to be below normative data.^19^

### Limitations

In considering the findings of this study, it is important to bear in mind the following limitations: only 26.5% of HCPs employed within this local municipality participated. While this is an adequate sample size for inferential statistics, it is not known whether the sample is representative of the whole group. Volunteer bias was likely to have occurred because of participants self-selecting whether they complete the questionnaire, as well as some participants being personally known to the principal investigator. This limitation may have excluded those HCPs with more severe burnout because of fear of stigma, discrimination or lack of support. The self-reporting nature of the questionnaire also could have introduced participant bias. Therefore, the results of this research may not be generalisable to other HCPs in South Africa.

The research sites may not have been a representative sample as participation was voluntary. Additionally, where field workers assisted in recruitment, participants were recruited from facilities, which the field workers could access easily using public transportation. The use of a cross-sectional design meant that causal inferences could not be made between individual and workplace factors with burnout. This made the interpretation of complex relationships between variables difficult. Technical limitations included the choice of the MBI as an instrument to measure burnout, as prior studies have not validated the MBI in healthcare workers in sub-Saharan Africa, and there may have been different cultural interpretations of questions related to the construct of burnout.^3^

### Recommendations

It is recommended that further research be conducted on burnout in a rural, district-level setting in the form of a qualitative study to better understand the nuances of the work environment. Performing longitudinal assessments of burnout along with measurements of mood, substance use, suicidality, cognition, performance and quality of life will add to the understanding of the burnout syndrome and its long-term consequences in a South African, rural, district-level setting.

It is further recommended that both evidence-based organisational and individual-focused solutions be explored and implemented to prevent burnout, with special consideration being given to improving work demands, managerial support and role clarity in rural, district-level settings as part of the effort to retain HCPs in the rural public health system.

## Conclusion

This research described the experience of burnout and examined associated individual and workplace factors among HCPs in a rural context in South Africa. Healthcare providers were found to experience high levels of burnout for Emotional Exhaustion and Personal Accomplishment but not for Depersonalisation. It was found that HCPs would seek help if they had an emotional problem, most likely from mental health professionals. Notwithstanding the limitations, this research is important in highlighting that improvements in work demands, managerial support and role clarity may reduce burnout through HCP experiencing increased personal accomplishment in the workplace.
